# Subchondral Phosphate Injection During Hip Arthroscopy Safely Treats Acetabular Bone Marrow Lesions in Early Osteoarthritis

**DOI:** 10.3390/jcm14238298

**Published:** 2025-11-22

**Authors:** Marco Minelli, Berardo Di Matteo, Vincenzo Longobardi, Alessio D’Addona, Marco Rosolani, Sebiano Pitronaci, Elizaveta Kon, Federico Della Rocca

**Affiliations:** 1 Department of Biomedical Sciences, Humanitas University, Via Rita Levi Montalcini 4, Pieve Emanuele, 20090 Milan, Italy; marcomariaminelli@gmail.com (M.M.); berardo.dimatteo@humanitas.it (B.D.M.); elizaveta.kon@humanitas.it (E.K.); 2 IRCCS Humanitas Research Hospital, Via Manzoni 56, 20089 Rozzano, Italy; alessio.daddona@humanitas.it (A.D.); marco.rosolani@humanitas.it (M.R.); federico.dellarocca@humanitas.it (F.D.R.); 3 Department of General Surgery and Medical Surgical Specialties, Section of Orthopedics and Traumatology, A.O.U. Policlinico-San Marco, University of Catania, Via Santa Sofia 78, 95123 Catania, Italy; sebianopitronaci@gmail.com

**Keywords:** subchondroplasty, calcium phosphate, acetabular, bone marrow, BML, hip, arthroscopy

## Abstract

**Introduction**: Arthroscopy for femoroacetabular impingement (FAI) yields inferior outcomes when subchondral edema and cystic degeneration are present. Subchondroplasty (SCP), which involves injecting osteoconductive calcium phosphate into Bone Marrow Lesions (BMLs), may enhance subchondral structural support and can be performed alongside hip arthroscopy. **Materials and Methods**: This single-center retrospective study included patients who underwent SCP for acetabular BMLs during primary hip arthroscopy for FAI from March 2019 to March 2023. Clinical and radiographic outcomes were recorded at ≥2-year follow-up. Survivorship with treatment failure as the endpoint was assessed using Kaplan–Meier analysis. **Results**: Thirty-four patients were evaluated at a mean 3.1-year follow-up. No perioperative, early, or late complications or adverse events occurred. No bone substitute migration or intra-articular extravasation was seen. Four patients (11.8%) showed osteoarthritis progression and required conversion to total hip arthroplasty; no intraoperative issues with acetabular preparation were encountered. All clinical scores improved significantly (*p* < 0.001), and 82.4% returned to sport. **Conclusions**: SCP performed during hip arthroscopy appears safe in selected patients with early hip osteoarthritis and BMLs. Calcium phosphate injection may help restore subchondral integrity and load distribution, though the independent contribution of SCP beyond standard arthroscopic management remains uncertain.

## 1. Introduction

Bone marrow lesions (BMLs) are localized areas of edema within subchondral bone, detected as low signal intensity areas on T1-weighted magnetic resonance imaging (MRI) and high signal intensity areas on T2-weighted fat-suppressed or fat-suppressed proton density MRI [1,2,3]. BMLs are suggestive of subchondral bone damage secondary to degenerative diseases such as osteoarthritis (OA) [1]. Subchondral acetabular edema and cystic degeneration are associated with increased pain, disability, and functional impairment [4]. When subchondral changes are associated with intra-articular pathologies, such as femoroacetabular impingement (FAI) and labral tears, arthroscopic treatment of these issues alone has been observed to provide inferior outcomes [5]. Histological analyses of bone marrow lesions have revealed features consistent with loss of mechanical integrity and non-healing chronic stress fractures [6]. In this scenario, a minimally invasive surgical technique known as subchondroplasty (SCP), which involves injecting an isothermic calcium phosphate solution into BMLs, has emerged as a potential treatment [7]. This material has osteoconductive properties and aims to provide structural support and to promote the remodeling and regeneration of the insufficient subchondral bone, potentially leading to improved pain and function [7]. SCP was initially developed for knee bone marrow lesions and showed significant improvements in pain and function with low complication rates when used in patients with mild-to-moderate knee osteoarthritis and persistent BMLs [8,9]. This procedure may be combined with hip arthroscopy to address concomitant intra-articular pathologies [10]. Subchondroplasty offers a minimally invasive option for treating acetabular cystic degeneration and bone marrow edema in the setting of concomitant intra-articular pathology. It may serve as a targeted intervention for selected patients with early hip osteoarthritis and preserved joint space, where advanced degeneration is absent. The primary endpoint of this study was to report perioperative, early, or late complications and adverse events of acetabular subchondroplasty, whenever present. The secondary endpoint was to evaluate survivorship, clinical, and radiographic outcomes of the SCP procedure at minimum two-year follow-up.

## 2. Materials and Methods

### 2.1. Study Design and Patients Selection

This is a monocentric retrospective study on a consecutive series of patients who underwent acetabular subchondroplasty to address bone marrow lesions during primary hip arthroscopy for femoroacetabular impingement from March 2019 to March 2023. All the surgeries were performed by a single high volume hip arthroscopy fellowship-trained orthopedic surgeon. The present study was approved by the independent Institutional Ethics Committee of IRCCS Humanitas Research Hospital (protocol number 618/17). Included patients underwent clinical and radiographic follow-up after a minimum of two years postoperatively. Inclusion criteria for acetabular subchondroplasty were as follows: (1) the presence of one or more acetabular BML(s) confirmed on T2-weighted fat-suppressed or fat-suppressed proton density MRI; (2) radiographic evidence of mild-to-moderate hip osteoarthritis (Kellgren–Lawrence (K-L) grade from 1 to 3 [11], Tonnis grade 1 or 2 [12]); (3) failed previous unsuccessful conservative non-surgical management (e.g., partial weightbearing, extracorporeal shockwave therapy, pulsed electromagnetic fields). Exclusion criteria encompassed the following: (1) MRI evidence of osteonecrosis in the affected compartment; (2) the presence of known systemic disorders or any systemic inflammatory condition (e.g., rheumatoid arthritis); (3) the presence of known metabolic bone disease; (4) severe osteoarthritis (Kellgren–Lawrence (K-L) grade 4, Tonnis grade 3); (5) hip dysplasia (lateral center-edge angle LCEA of Wiberg < 25° and Tonnis angle > 10° [13,14]).

### 2.2. Surgical Procedure

**Figure 1:**
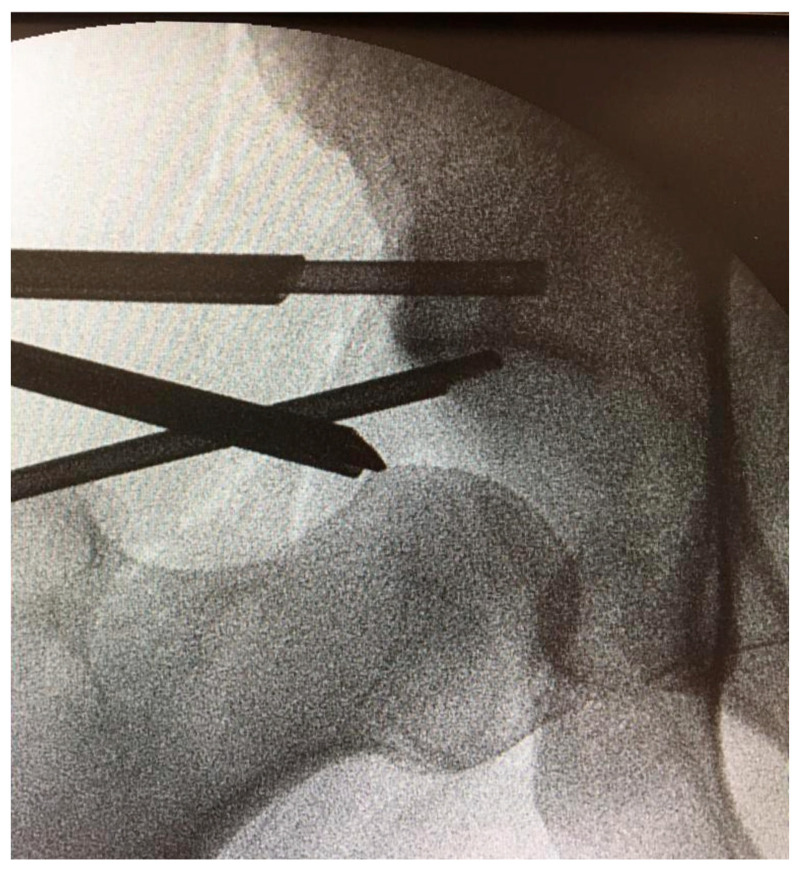
Bone marrow lesion targeting for correct AccuFill^®^ injection by using orthogonal fluoroscopic views that matched the associated MRI sliced.

**Figure 2:**
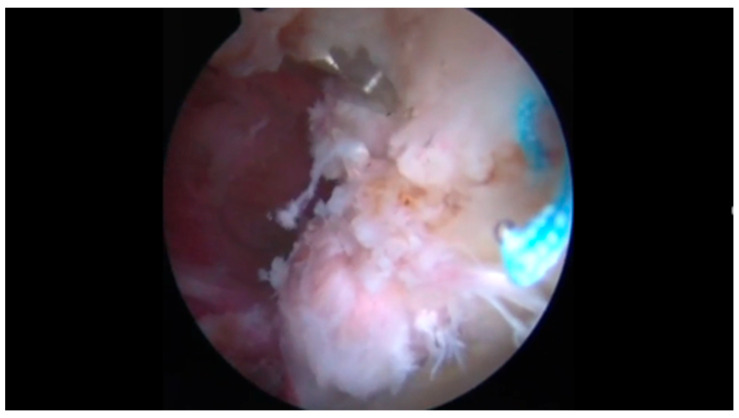
The fluid bone substitute was injected into the cyst under fluoroscopic guidance and intra-articular visualization to prevent cartilage damage with the cannula and avoid intra-articular extravasation.

Preoperatively, a standard anteroposterior pelvis and a modified Dunn [15] view hip radiograph, as well as an MRI arthrogram were requested for every patient as diagnostic work-up for intra-articular pathologies. Preoperative MRI was used to determine the location of the bone marrow lesions relative to radiographic landmarks and to plan the access point, the trajectory, and depth of dedicated cannulas. This information was used intraoperatively to target the bone defect for correct AccuFill^®^ (Zimmer Knee Creations Inc., Exton, PA, USA; manufactured by Etex Corporation, Braintree, MA, USA) injection by using orthogonal fluoroscopic views that matched the associated MRI slices. Spinal loco-regional anesthesia was administered prior to the procedure. Patients were positioned supine on a traction table (Advanced Supine Hip Positioning System; Smith & Nephew, Sesto San Giovanni, Milan, Italy) with a padded perineal post to avoid pudendal nerve and genital injuries. The operative limb was placed in neutral flexion and extension, 10° to 15° of adduction, and 20° to 30° of foot internal rotation to achieve adequate distraction allowing for access to the intra-articular compartment. Traction was then gently applied to the limb until 8–10 mm of distraction was obtained and confirmed on fluoroscopic imaging. Access to the hip joint was obtained through the anterolateral, distal anterolateral, and mid-anterior arthroscopic portals; a 70° arthroscope (Smith & Nephew) was used. Diagnostic arthroscopy was performed to evaluate overall chondral damage (which was classified according to the Outerbridge classification [16]) and chondrolabral complex lesions (classified according to Acetabular Labrum Articular Disruption ALAD classification [17]). Chondral lesions were stabilized and acetabular labrum was debrided whenever necessary by using a 4.5 mm shaver (DYONICS Shaver; Smith & Nephew), then labral tears were repaired through an appropriate number of resorbable 1.8 mm all-suture anchors (Q-FIX All-Suture Anchor; Smith & Nephew). If a small irreparable labrum lesion was present, a partial labral resection was performed. A labral reconstruction by using an ilio-tibial band autograft was carried out in case of large non-repairable labrum tears which were considered to have significant functional impact. No other accessory procedure was performed on the cartilage. Whenever a pincer morphology was present, an extra-articular acetabular trimming was performed by using a high-speed 5.5 mm burr (DYONICS Burr; Smith & Nephew) before addressing intra-articular labral issues. The AccuPort^®^ cannula (Zimmer Knee Creations, West Chester, PA, USA) was drilled through the anterolateral portal into the subchondral bone cyst with a wire driver under fluoroscopic guidance: targeting was achieved by centering the targeted defect in the beam and aligning the wire driver and AccuPort^®^ cannula with the beam while drilling (Figure 1). This was performed under direct intra-articular visualization to prevent cartilage damage with the cannula (Figure 2). AccuFill^®^ is a bone graft substitute that resorbs and is replaced with new bone during the healing process. AccuFill^®^ is composed of 2 engineered calcium phosphate (CaP) forms (amorphous CaP and dicalcium phosphate dihydrate). As soon as it is injected, AccuFill^®^ rapidly interdigitates into the subchondral bone defects and undergoes an isothermal reaction at body temperature to set hard and avoid thermal necrosis. AccuFill^®^ is hydrated and mixed before injection, using normal saline (0.9%), and releases carbon dioxide during the setting process to form pores within the material. AccuFill^®^ was mixed using the AccuMix^®^ mixing system, which provides closed mixing of AccuFill^®^, and transferred to injection syringes with a standard Luer-lock connection. The result is a nanocrystalline, macroporous, and osteoconductive scaffold with physical properties similar to cancellous bone and a chemical formulation and 3-dimensional structure suitable for bone mechanical support and cell-mediated remodeling [18]. The inner stylus of the cannula was removed, and the fluid bone substitute was injected into the cyst under fluoroscopic guidance and intra-articular visualization through the camera until back pressure was felt in the syringe to avoid overfilling of the cyst. The stylus was reinserted into the cannula and left in place for approximately 10–12 minutes so that the bone graft substitute could crystallize and harden through an isothermal reaction that occurs only at body temperature. If extravasation of the bone substitute into the hip joint occurred, this material was debrided and removed with the shaver. The cannula was then retrieved and traction was released. Whenever a cam lesion was present, capsulotomy was first performed with radiofrequencies (VAPR VUE Radiofrequency Electrode System, Johnson & Johnson MedTech, Pratica di Mare, Pomezia Roma, Italy), then femur neck was reshaped under fluoroscopic guidance via a DYONICS 5.5 mm high-speed burr. Dynamic tests were performed intraoperatively to check for residual impingement. The capsule was then closed with non-absorbable sutures. Immediate postoperative partial weightbearing was allowed for every patient and was maintained for one month. Anteroposterior pelvis radiographs were obtained immediately after the procedure and at the one-month postoperative follow-up examination to verify proper filling of the acetabular cyst. Then, included patients underwent clinical and radiographic follow-up three and six months and every year postoperatively.

### 2.3. Patients Evaluation

Any perioperative, early or late complications, adverse events, and/or treatment failures were reported. A serious adverse event was defined as an adverse event leading to a death, injury, or permanent impairment to a body structure or a body function. A minor adverse event was defined as the presence of tenderness, redness, and edema of the index hip at the first follow-up visit. Treatment failure was defined as any further treatment performed or prescribed (both injective or surgical) on the index hip due to persistent or worsening symptoms after the procedure. Clinical evaluation was performed preoperatively and postoperatively and included return to sport, the modified Harris Hip Score (mHHS), the International Hip Outcome Tool 12 (iHOT-12), and the Visual Analog Scale (VAS) [19,20,21]. At the last follow-up, radiographic follow-up was performed on a standard anteroposterior pelvis radiograph and a modified Dunn view hip radiograph. Bone substitute migration, intra-articular extravasation, and/or radiographically evident local complications were reported whenever present. Kellgren–Lawrence and Tonnis grades were calculated to evaluate osteoarthritis evolution over time. All the images were evaluated by two orthopedic specialists (A.D. and M.R.) trained in hip surgery. Inter-reader reliability was assessed using Cohen’s kappa, which demonstrated excellent agreement (κ = 0.90).

### 2.4. Statistical Analysis

Kaplan–Meier methodology was used to estimate survivorship, with treatment failure defined as the endpoint. For patients who could not be contacted further or were deceased without an exact date of death, their most recent documented follow-up was used for censoring. Numbers-at-risk at each time point were included in the Kaplan–Meier survival curve to facilitate interpretation of survival probability over time. Continuous variables were summarized using means with standard deviations or reported as ranges (minimum–maximum), whereas categorical variables were presented as counts and percentages. Rates of complications and treatment failures were calculated as crude proportions. Normality was assessed with the D’Agostino–Pearson test; depending on data distribution, paired *t*-tests or Mann–Whitney tests were applied to compare groups. Categorical comparisons were performed using chi-square or Fisher’s exact tests where appropriate. Effect sizes (Cohen’s d) were calculated for changes in mHHS, iHOT-12, and VAS. Clinically meaningful improvement was assessed by comparing postoperative improvements with the published minimal clinically important difference (MCID) for each PROM. MCID thresholds were defined as mHHS = 8–10 points [22], iHOT-12 ≈ 13 points [23], VAS ≈ 1.5 points [24]. Ninety-five percent confidence intervals (95% CI) were reported where applicable. Statistical analysis was performed with EasyMedStat software (version 3.42; www.easymedstat.com; accessed on 27 April 2025).

## 3. Results

### 3.1. Patients Characteristics

A total of 34 patients underwent acetabular subchondroplasty during the index period. No patient was lost to a minimum two-year follow-up. Thus, 34 patients were reviewed clinically and radiographically at a mean follow-up of 3.1 years (range 2.0–6.0 years). Patients comprised 30 males (88.2%) and 4 females (11.8%). Mean age at surgery was 37.4 years (range 23.0–53.0). Mean BMI was 24.5 (range 19.1–29.5). Mean surgical time was 98.0 min (range 55–188 min), and mean traction time was 40.4 min (range 25.0–89.1 min). A summary of patients’ characteristics and surgical procedures is reported in Table 1 and Table 2.

### 3.2. Complications, Adverse Events and Failures

**Figure 3:**
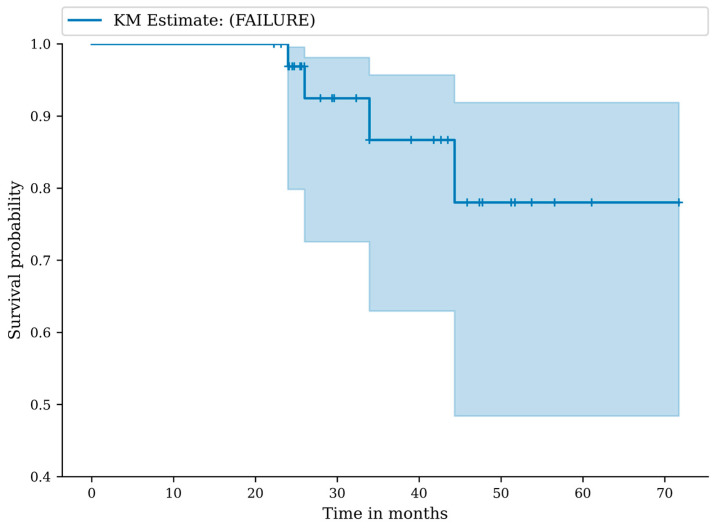
Kaplan–Meier (KM) survival curve with any failure of the procedure as endpoint.

Follow-up rate was 100%. The median duration of follow-up was 30 months (22–71 months). No perioperative, early, or late complications were reported. No serious or minor adverse events were recorded. We recorded a total of four failures (11.8%). At 12 months, the failure-free survival was 100.0% (95% CI: 100.0–100.0), at 24 months the failure-free survival was 96.9% (95% CI: 79.8–99.6), and at 36 months the failure-free survival was 86.7% (95% CI: 63.0–95.7) (Figure 3). Numbers-at-risk at 0, 12, 24, 36, 48, and 60 months were 34, 34, 33, 30, 30, and 30, respectively. In particular, four of the treated patients were scheduled for total hip arthroplasty at the last follow-up because of severe hip osteoarthritis (K-L grade 4 and Tonnis grade 3). These patients presented an intraoperative Outerbridge grade 4 chondral damage during the index procedure. A standard hemispheric titanium shell was used for these patients, and no intraoperative complications in acetabular reaming and cup positioning were observed during total hip arthroplasty procedure.

### 3.3. Clinical Outcomes

All the clinical scores significantly improved at the last follow-up compared to preoperative status. Preoperative mean mHHS was 65.9 (range 52.0–73.0); postoperative mean mHHS was 93.1 (range 66.0–100.0), *p* < 0.001. Preoperative mean iHOT-12 was 40.5 (range 30.0–56.5); postoperative mean iHOT-12 was 88.5 (range 24.3–100.0), *p* < 0.001. Preoperative mean VAS was 4.9 (range 3.0–8.0); postoperative mean VAS was 1.9 (range 0–7.0), *p* < 0.001. All PROMs exceeded MCID thresholds. The mean ΔmHHS improvement was +27.2 points (95% CI, 24.27–30.13), exceeding the MCID threshold of 8–10 points. The mean ΔiHOT-12 improvement was +48.0 points (95% CI, 41.68–54.32), exceeding the MCID threshold of 13 points. The mean ΔVAS improvement was −3.0 points (95% CI, −3.69 to −2.31), exceeding the MCID threshold of ~1.5 points. Effect-size analysis further demonstrated very large clinical benefit: mHHS improved with a Cohen’s d of 3.15 (95% CI: 2.43–3.86), iHOT-12 improved with a Cohen’s d of 3.02 (95% CI: 2.32–3.72), and VAS decreased with a Cohen’s d of −1.73 (95% CI: −2.29 to −1.17). Twenty-eight patients returned to their preinjury sport after the procedure: return to sport rate was 82.4%.

### 3.4. Radiographic Outcomes

**Figure 4:**
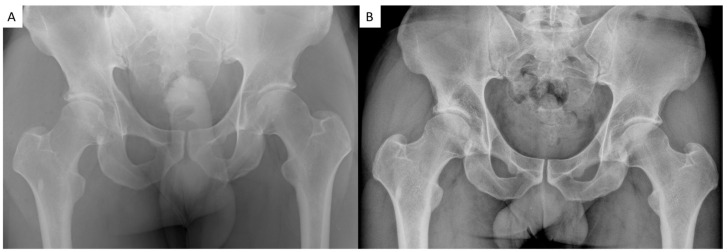
(**A**): immediate postoperative radiographic control; (**B**): 5-year radiographic follow-up showing no bone substitute migration, intra-articular extravasation, and/or local complications.

**Figure 5:**
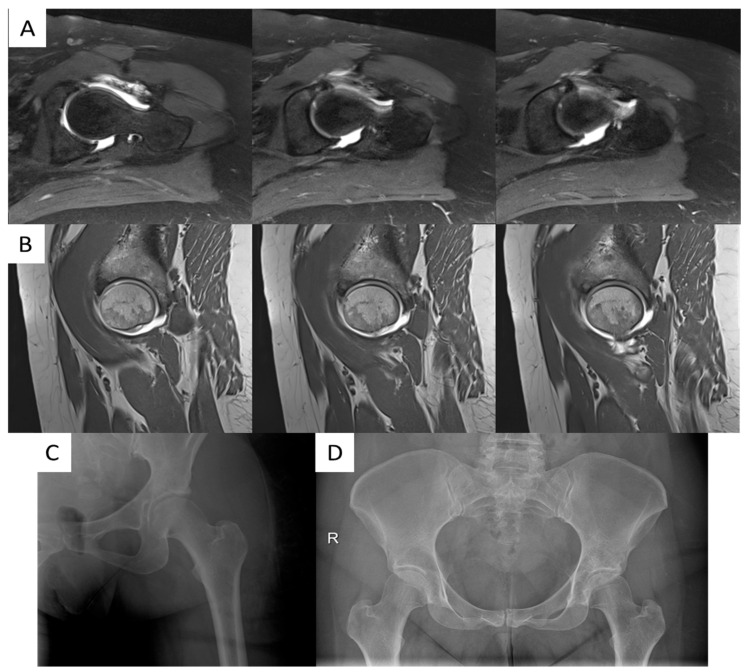
(**A**): preoperative T2-weighted MR arthrogram axial views showing antero-superior labral tear and acetabular subchondral bone cystic degeneration; (**B**): preoperative T1-weighted MR arthrogram sagittal views showing antero-superior labral tear and acetabular subchondral bone cystic degeneration; (**C**): immediate postoperative radiographic control; (**D**): 4-year radiographic follow-up. R: right.

At radiographic evaluation, bone substitute migration, intra-articular extravasation, and/or radiographically evident local complications were not observed (Figure 4 and Figure 5). Radiographic evidence of osteoarthritis progression over time was observed only in the four patients who were scheduled for total hip arthroplasty (11.8%). In particular, preoperative Kellgren–Lawrence was grade 1 for 5 patients (14.7%), grade 2 in 21 cases (61.8%), and grade 3 for 8 patients (23.5%); postoperative Kellgren–Lawrence was grade 1 for 5 patients (14.7%), grade 2 in 20 cases (58.8%), grade 3 for 5 patients (14.7%) and grade 4 in 4 cases (11.7%). Preoperative Tonnis was grade 1 for 23 patients (67.6%) and grade 2 in 11 cases (32.4%); postoperative Tonnis was grade 1 for 23 patients (67.6%), grade 2 in 7 cases (20.6%) and grade 3 for 4 patients (11.7%).

## 4. Discussion

To date, this is the first study investigating subchondroplasty for acetabular cystic degeneration and bone marrow edema in patients undergoing hip arthroscopy for femoroacetabular impingement. The main finding of the study is that acetabular subchondroplasty combined with hip arthroscopy appears to be a safe procedure in selected patients with early hip osteoarthritis and maintained joint space and does not present complications at minimum two-year follow-up. Promising clinical and radiographic outcomes were observed, but the specific contribution of subchondral bone substitute injections versus femoroacetabular impingement treatment in yielding these outcomes is unclear: subchondroplasty could represent a therapeutic solution in the case of acetabular bone marrow lesions, but further comparative studies are needed.

No perioperative or late complications were reported, and no adverse events were recorded. None of the patients reported presence of tenderness, redness, and edema of the index hip at the first follow-up visit. This could be facilitated by the fact that acetabular subchondroplasty is a fast procedure: mean traction time was about 40 minutes. Moreover, attention was paid to avoid overfilling of the cysts, which was described to cause immediate postoperative pain [7]. Plus, bone substitute migration, intra-articular extravasation, and/or local complications were never observed at radiographic evaluation. Similarly, subchondroplasty was observed to be a safe procedure for the treatment of symptoms related to persisting BMLs in mild-to-moderate osteoarthritic knees [8]. Indeed, subchondroplasty is a minimally invasive procedure, and AccuFill^®^ is a bone graft substitute composed of calcium phosphate, which is a biocompatible material that interdigitates with bone for complete defect fill and undergoes an isothermal reaction at body temperature, avoiding thermal necrosis [14]. Then, AccuFill^®^ is gradually resorbed and replaced by natural bone and, thus, minimizing risks of adverse reactions over time [14]. Bone substitute leakage outside the targeted site was described as a subchondroplasty complication [8,25]. In this series, direct intra-articular arthroscopic visualization prevented cartilage damage with the cannula and avoided intra-articular material leakage.

In this cohort, significant improvements were observed in the clinical scores and return to preinjury sport rate was 82.4% at a median follow-up of 30 months. Likewise, a meta-analysis reported an 84.6% return to sport rate after hip arthroscopy at a mean follow-up of 25.8 ± 2.4 months [26]. This was true even if the cohort’s mean patient age was 37.4 years, and increasing age was observed to be associated with worse outcomes after hip arthroscopy for femoroacetabular impingement [27,28]. Moreover, all the included patients were diagnosed with mild-to-moderate osteoarthritis, and increasing severity of osteoarthritis was observed to influence hip arthroscopy postoperative outcomes and survivorship: the rate of hip arthroscopy conversion to total hip arthroplasty was reported to range from 9.5% to 50% in the case of osteoarthritis [29,30]. In particular, Daivajna et al. observed that 44% of patients with advanced osteoarthritis required total hip arthroplasty at a mean of 18 months after hip arthroscopy for femoroacetabular impingement [31]. In patients with mild-to-moderate Tonnis grade 1 and 2 osteoarthritis, a 13% failure rate was recorded [32]. Similarly, the failure rate in this cohort was 11.8%.

All the clinical scores significantly improved at the last follow-up compared to preoperative status: mean postoperative mHHS in this cohort was 93.1 and mean VAS was 1.9. Accordingly, Huang et al. reported a mean postoperative mHHS of 88.82 ± 11.60 and a mean VAS of 1.93 ± 1.89 in a cohort of 159 patients after hip arthroscopy for femoroacetabular impingement [33]. It is unclear whether the outcomes observed in this cohort were secondary to the intra-articular pathologies management or to the acetabular bone substitute injections. However, arthroscopic treatment of intra-articular pathologies, such as femoroacetabular impingement (FAI) and labral tears, was reported to provide inferior outcomes whenever subchondral changes were not addressed [5]. Indeed, Krych et al. observed mean mHHS to be inferior for patients with subchondral edema/cystic change when compared to those without subchondral bone damage (79.9 ± 18.7 vs. 86.6 ± 12.5; *p* = 0.03) [5]. The presence of BMLs correlates with pain and rapid joint deterioration: bone marrow lesions represent biomechanical and histological subchondral bone alterations [34,35,36]. Bone marrow lesions occur whenever physiologic subchondral remodeling fails because of ongoing joint forces, increased stress focalization, and histologically represent non-healing chronic stress fractures of subchondral bone [37,38]. Bone marrow lesions predict loss of integrity of the subchondral bone contributing to future collapse and deformity of the joint [39]. Thus, interventions aimed at repairing subchondral bone defects could relieve symptoms and alter the progression of joint deterioration. In this scenario, subchondroplasty could be a therapeutic option, since AccuFill^®^ has physical properties similar to cancellous bone and a chemical formulation and three-dimensional structure suitable for bone mechanical support and cell-mediated remodeling [14]. Thus, AccuFill^®^ could provide subchondral bone repair by providing mechanical support and cell-mediated remodeling [14]. By filling the BMLs, subchondroplasty helps restore the structural integrity of bone, thereby improving load distribution across the joint: this reduces abnormal stress concentrations that can lead to further cartilage degradation, which is a key factor in osteoarthritis progression [40,41]. This procedure could also increase the local stiffness of the bone, restoring subchondral bone mechanical behavior and reducing the risk of subchondral fracture or further collapse. Moreover, by providing a scaffold for bone to remodel and heal, subchondroplasty could promote the biological repair of damaged subchondral bone, which may further aid in the restoration of joint function. Calcium phosphate materials were demonstrated to have osteoconductive properties, supporting bone growth and integration [42,43]. In fact, a standard hemispheric titanium shell was enough to achieve appropriate intraoperative press-fit stability, and no intraoperative complications in acetabular reaming and cup positioning were observed in patients who required a conversion to total hip arthroplasty. As the injected bone substitute gradually resorbs and is replaced by bone tissue, the long-term biomechanical properties of the joint could improve [43]. However, further studies are needed in order to describe biomechanical and histological behavior of acetabular subchondral bone after calcium phosphate injections over time. Nonetheless, radiographic evidence of osteoarthritis progression over time was observed only in patients who were scheduled for total hip arthroplasty. All of these patients were diagnosed with a Tonnis grade 2 osteoarthritis on preoperative radiographs, and an Outerbridge grade 4 chondral damage was observed intraoperatively. Indeed, intraoperative cartilage degeneration was described to predict poorer outcomes after hip arthroscopy: individuals with grade 4 chondral defects exhibit poorer functional outcomes, lower satisfaction, and increased pain levels compared to those with less severe chondral damage or no chondral injury [44,45]. These findings reinforce that imaging biomarkers and intra-articular cartilage status correlate with prognosis, underscoring the importance of patient stratification and selection to guide interventions within a personalized medicine framework. However, this study lacks a control group: to confirm our findings, a comparative clinical and radiographic evaluation involving gender-, age-, and intra-articular pathology-matched patients with acetabular bone marrow lesions not undergoing subchondroplasty/undergoing other procedures, such as subchondral perforations, would be needed. This strongly limits the reliability of our conclusions.

This study presents other limitations. First, as SCP was performed in combination with hip arthroscopy, improvements in clinical outcomes must be interpreted cautiously: concomitant procedures—including labral repair and correction of FAI morphology—represent confounders that limit our ability to determine the specific contribution of SCP. Therefore, these findings better support its safety and technical feasibility rather than definitive clinical superiority over arthroscopy alone. The retrospective design limits the strength of evidence and precludes detailed assessment of how clinical and radiographic findings evolved over time. Moreover, the small cohort of included patients leads to a lower statistical power, and individual data points could have a disproportionate impact on the results: a single outlier or unique data point might either obscure or falsely suggest a trend that would not exist in a larger group. Furthermore, reporting crude event proportions may underestimate the true event burden, as some events may not have occurred by the latest follow-up and, therefore, would not be captured. Finally, the lack of postoperative MRI data prevented us from documenting eventual subchondral bone remodeling, bone marrow lesions resolution, or chondral damage progression following subchondroplasty.

## 5. Conclusions

Acetabular subchondroplasty performed in conjunction with hip arthroscopy was safe and feasible in a carefully selected cohort with femoroacetabular impingement, early osteoarthritis, and MRI-defined acetabular bone marrow lesions, demonstrating no perioperative or late complications and significant improvements in patient-reported outcomes. Importantly, the inclusion/exclusion criteria operationalize a personalized medicine approach: intervention guided by imaging biomarkers (BMLs, preserved joint space) and intra-articular cartilage status (avoiding advanced degeneration) and correlated with prognosis. These findings support SCP as a targeted, minimally invasive option for a defined patient phenotype, potentially improving load distribution and subchondral integrity while preserving future arthroplasty options. Given the study’s retrospective design, small sample size, and absence of postoperative MRI, prospective comparative trials are warranted to validate patient-selection thresholds, quantify the incremental benefit of SCP over arthroscopy alone, and evaluate imaging-based response predictors to further refine precision care in hip preservation.

## Figures and Tables

**Table 1:** Patients’ characteristics.

Patient	Gender	Age at Surgery	BMI	Preoperative Tonnis	Preoperative Kellgren–Lawrence
P01	F	29.0	19.13	1	1
P02	M	35.0	22.72	1	2
P03	M	40.0	25.26	1	2
P04	M	41.0	26.59	1	3
P05	F	25.0	24.09	1	1
P06	M	37.0	26.3	1	2
P07	M	38.0	29.54	1	2
P08	F	28.0	24.22	1	2
P09	M	23.0	22.86	2	2
P10	M	38.0	24.38	1	2
P11	M	28.0	24.58	1	2
P12	M	33.0	27.14	1	2
P13	M	35.0	24.58	1	2
P14	M	46.0	25.9	2	3
P15	M	53.0	22.22	2	3
P16	M	43.0	23.55	2	2
P17	M	46.0	25.93	1	2
P18	M	36.0	24.65	2	3
P19	M	42.0	26.96	1	1
P20	M	47.0	26.22	2	3
P21	M	50.0	22.2	1	2
P22	F	50.0	22.2	1	2
P23	M	39.0	27.47	1	1
P24	M	33.0	26.3	1	1
P25	M	26.0	25.98	2	3
P26	M	50.0	24.34	2	2
P27	M	32.0	27.13	1	2
P28	M	23.0	23.29	1	2
P29	M	26.0	26.78	2	2
P30	M	29.0	21.47	2	3
P31	M	29.0	21.47	2	3
P32	M	49.0	24.58	1	2
P33	M	46.0	20.94	1	2
P34	M	46.0	20.94	1	2

**Table 2:** Surgical procedures.

Patient	Surgical Time (min)	Acetabular Trimming	Anterior Superior Partial Labral Resection	Labral Reconstruction	Femur Neck Reshaping	Bone Substitute Injected (cc)	Acetabulum Intraoperative Grade (I–IV)
P01	188	no	no	no	yes	3	1
P02	65	no	no	no	yes	3	4
P03	77	no	no	no	yes	3	4
P04	80	no	yes	no	yes	3	3
P05	80	no	no	no	yes	3	2
P06	112	no	yes	yes	yes	3	3
P07	104	no	no	no	yes	3	3
P08	95	no	no	no	yes	9	4
P09	114	no	no	no	yes	15	4
P10	108	no	no	no	yes	3	4
P11	115	no	no	no	yes	15	4
P12	85	no	no	no	yes	9	4
P13	107	yes	no	no	yes	3	2
P14	89	no	no	no	yes	3	4
P15	98	no	yes	no	yes	3	3
P16	79	no	no	no	yes	3	3
P17	115	no	no	no	yes	3	2
P18	83	no	yes	no	yes	2.5	4
P19	97	no	no	no	yes	3	3
P20	108	yes	no	no	yes	3.5	4
P21	114	no	yes	no	yes	3	3
P22	114	no	yes	no	yes	3	3
P23	115	no	no	no	yes	3	2
P24	81	no	no	no	yes	3	3
P25	47	no	yes	no	yes	3	3
P26	109	no	no	no	yes	3	3
P27	109	no	no	no	yes	3	3
P28	98	no	no	no	yes	3	4
P29	110	no	no	no	yes	3	4
P30	55	yes	yes	no	yes	3	4
P31	55	yes	yes	no	yes	3	2
P32	106	no	no	no	yes	3	4
P33	110	yes	no	no	yes	3	4
P34	111	yes	no	no	yes	3	4

## Data Availability

The original contributions presented in this study are included in the article. Further inquiries can be directed to the corresponding author.
